# AKT isoform-specific expression and activation across cancer lineages

**DOI:** 10.1186/s12885-018-4654-5

**Published:** 2018-07-16

**Authors:** Jue Wang, Wei Zhao, Huifang Guo, Yong Fang, Sarah Elizabeth Stockman, Shanshan Bai, Patrick Kwok-Shing Ng, Yang Li, Qinghua Yu, Yiling Lu, Kang Jin Jeong, Xiaohua Chen, Meng Gao, Jiyong Liang, Wentao Li, Xingsong Tian, Eric Jonasch, Gordon B. Mills, Zhiyong Ding

**Affiliations:** 1 Department of Breast and Thyroid Surgery, Shandong Provincial Hospital Affiliated to Shandong University, Jinan, Shandong China; 2 Department of Systems Biology, The University of Texas MD Anderson Cancer Center, 1515 Holcombe Blvd, Houston, TX 77030 USA; 3 Genitourinary Medical Oncology, The University of Texas MD Anderson Cancer Center, Houston, TX 77030 USA; 4 Institute for Personalized Cancer Therapy, The University of Texas MD Anderson Cancer Center, Houston, TX 77030 USA; 5 Department of Interventional Radiology, Cancer Hospital, Fudan University, Shanghai, 200032 China

**Keywords:** AKT, Isoform, Expression, Phosphorylation, Activation, RPPA

## Abstract

**Background:**

Aberrant AKT activation is prevalent across human cancer lineages, providing an important therapeutic target. AKT comprises three isoforms that mediate critical non-redundant, even opposing functions in cancer pathophysiology. Therefore, targeting specific AKT isoforms in particular cancers may be more effective than pan-AKT inhibition while avoiding disadvantages of pan-AKT inhibition. Currently, AKT isoform-specific expression and activation in cancer are not clearly characterized.

**Methods:**

We systematically characterized AKT isoform-specific expression and activation in 211 cancer cell lines derived from different lineages and genetic backgrounds using a reverse-phase protein array platform.

**Results:**

We found that phosphorylation, but not expression, of AKT1 and AKT2 was coordinated in most but not all cells. Different cancer lineages displayed differential AKT1 and AKT2 expression and phosphorylation. A PIK3CA hotspot mutation H1047R but not E545K was associated with selective activation of AKT2 but not AKT1.

**Conclusions:**

Our study identified and validated AKT isoform-specific expression and phosphorylation in certain cell lines and demonstrated that genetic changes can affect AKT isoform-specific activation. These results provide a more precise understanding of AKT isoform-specific signaling and, in addition, facilitate AKT isoform targeting for personalized cancer therapies.

**Electronic supplementary material:**

The online version of this article (10.1186/s12885-018-4654-5) contains supplementary material, which is available to authorized users.

## Background

The serine/threonine kinase AKT (also known as protein kinase B, or PKB) plays critical roles in many aspects of cancer pathophysiology, including cell survival, growth, metabolism, and metastasis [1–3]. AKT is frequently activated in cancer through a variety of mechanisms, including amplification of growth factor receptors (e.g., HER2/neu and EGFR), amplification or mutation of phosphatidylinositol 3-kinase (PI3K), amplification or mutation of AKT isoforms, and inactivation of phosphatase and tensin homolog (PTEN) or inositol polyphosphate-4-phosphatase type II (INPP4B) or VHL [3, 4]. Therefore, AKT has been extensively explored as an anticancer therapeutic target [5]. However, since AKT controls many normal cellular functions [6], pharmacologic inhibition of all AKT isoforms could compromise the functions of normal cells and cause severe side effects in patients [7, 8], which is one likely reason that AKT inhibitors have been less successful than expected in the clinic.

AKT comprises three isoforms (AKT1, AKT2, and AKT3), and the different isoforms are believed to mediate critical non-redundant or even opposing functions in cancer pathophysiology [9–16]. For example, AKT1 has been demonstrated to suppress while AKT2 promotes breast cancer cell migration and invasion in vitro [9, 17, 18]. The opposing functions of AKT1 and AKT2 in cell migration and invasion were also demonstrated in vivo in mouse models. Similarly, AKT1 activation decreases tumor metastatic dissemination but promotes mammary tumorigenesis in mouse models, whereas AKT2 primarily increases tumor metastasis in those models [10–12]. Moreover, specific AKT isoforms have been demonstrated to be drivers in particular cancers. For example, AKT2, but not AKT1, mediates survival and maintenance of PTEN-deficient prostate cancer [19]. Furthermore, activating mutations in AKT1 are much more common than those in AKT2 or AKT3 with for example the E17K activating mutation in the PH domain being more than 25 fold less frequent in AKT2 or AKT3 than in AKT1. Therefore, inhibition of specific AKT isoforms in particular cancers at specific stages provides an approach that could be used to target the effects of cancer drivers and would overcome the disadvantages of pan-AKT inhibition in terms of toxicity. Currently, the complexities of AKT isoform-specific expression and activation are not clearly understood. The purpose of this study was to systematically characterize AKT isoform-specific expression and activation in cancer cells from different lineages and genetic backgrounds. This characterization will further improve our understanding of AKT isoform-specific signaling and facilitate the development of therapeutic approaches to targeting AKT isoforms for personalized and precision cancer therapies.

We characterized a set of AKT antibodies and used a reverse-phase protein array (RPPA) platform to analyze AKT isoform-specific expression and activation phosphorylation in 211 cell lines across different lineages. Our results revealed that AKT1 and AKT2 isoforms have distinct expression patterns and are differentially activated in certain cell lines.

## Methods

### Cell culture

The parental, AKT1−/−, AKT2−/−, and AKT1/2−/− double knockout (DKO) HCT116 colon cancer cell lines were gifts from Dr. Bert Vogelstein (Johns Hopkins University, Baltimore, MA). MCF10A cells expressing PIK3CA E545K or H1047R were gifts from Dr. Joan S. Brugge (Harvard Medical School, Boston, MA). Two endometrial adenocarcinoma cell lines HEC151 and HEC50 and an ovarian cancer cell line HOC8 were from MD Anderson Characterized Cell Line Core (Houston, TX). HCT116 cells were cultured in McCoy’s 5A Medium from Gibco (Carlsbad, CA). MCF10A cells were cultured as previously described [20]. The 211 cell lines were cultured in MD Anderson RPPA core (Additional file 1). Cell line identity was routinely confirmed via short tandem repeat profiling in the MD Anderson Characterized Cell Line Core.

### Antibodies

Antibodies against pan-AKT (#4691), phospho-AKT Thr308/9 (#2965), phospho-AKT Thr450/1 (#9267), phospho-AKT Ser473/4 (#9271), AKT1 (#2938), pAKT1 Ser473 (#9018), AKT2 (#3063), phospho-AKT2 (pAKT2) Ser474 (#8599), AKT3 (#14982), and ERK (#4695) were from Cell Signaling Technology (Beverly, MA).

### Lysate preparation

To prepare cell lysates for Western blot analysis, we lysed cells in radioimmunoprecipitation assay (RIPA) buffer (50 mM Tris-HCl [pH 7.4], 150 mM NaCl, 1% NP-40, 0.5% sodium deoxycholate, and 0.1% sodium dodecyl sulfate), supplemented with protease inhibitor and phosphatase inhibitor cocktails (Pierce Biotechnology, Rockford, IL). A Western blot analysis was performed as described previously [21].

### Statistical analyses

Key experiments were performed at least three times. Representative Western blots are presented. For quantification data presented with error bars, the means of data from triplicate repeats are presented with the standard deviations as error bars.

## Results

### Characterization of isoform-specific antibodies for pan-AKT and phospho-AKT

**Fig. 1:**
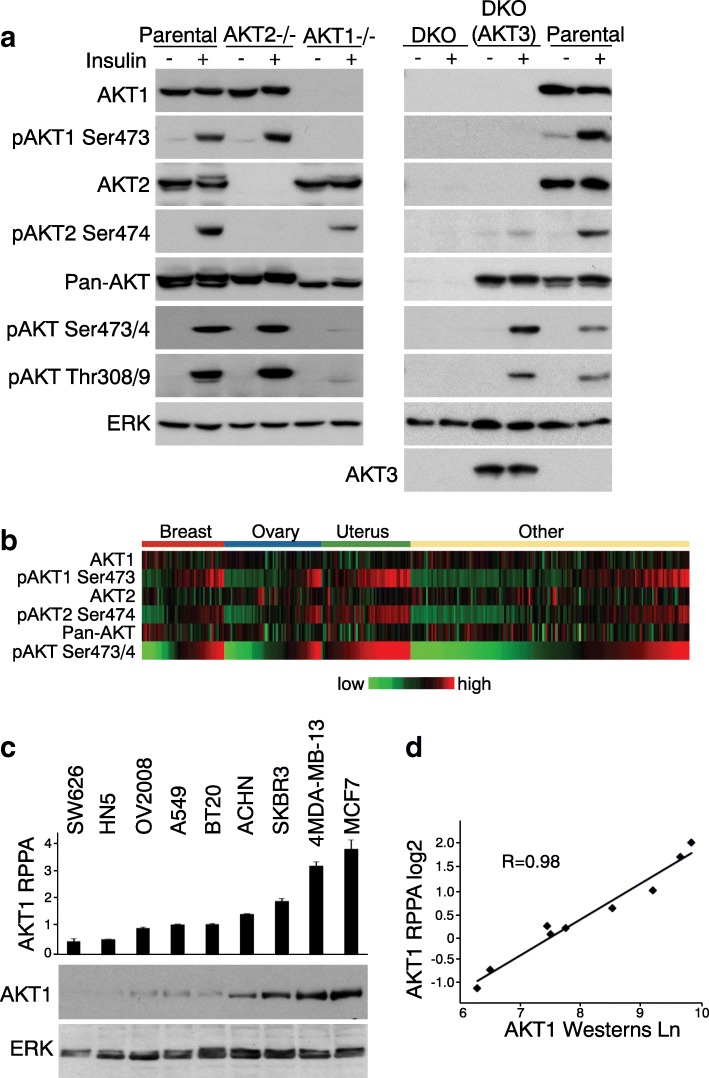
Validation of AKT isoform-specific antibodies **a** Validation of AKT isoform-specific antibodies by Western blotting. HCT116 parental, AKT2−/−, AKT1−/−, DKO, and DKO transfected with AKT3 cells were serum starved overnight and treated (or not treated) with insulin (20 μg/ml) for 30 min. Cells were lysed in RIPA buffer with protease inhibitors and phosphatase inhibitors. Lysates (50 μg/lane) were resolved in 10% sodium dodecyl sulfate polyacrylamide gel electrophoresis (SDS PAGE). Antibodies for each blot are listed to the left. ERK immunoblotting showed equivalent loading. **b** Heat map of RPPA features of protein and phospho-protein levels of AKT1, AKT2, and pan-AKT across 211 cell lines. Red, higher expression (relative to median across all cancers); green, lower expression. Cell lines are ordered by low to high pAKT Ser473/4 in each group. **c** Validation of AKT1 isoform-specific antibody for RPPA. Lysates of 211 cell lines were analyzed by RPPA platform in triplicate using AKT1 antibody. Selected cell lines expressing AKT1 at low to high levels per RPPA data are shown in the bar graph, with standard deviations as error bars. AKT1 levels in the selected cell lines (20 μg total protein/lane) were examined by western blotting. ERK immunoblotting was used as loading indicator. Scanning densitometric values of AKT1 on western blotting were obtained using ImageJ software (version 1.46r; National Institutes of Health, Bethesda, MD). **d** Correlation coefficient between AKT1 signals derived from RPPA (Y axis) and western blotting (X axis). RPPA data are presented as log2-transformed values. Western data presented as ln-transformed densitometric values

To analyze AKT isoform-specific expression and activation, we first characterized AKT specific antibodies using a set of parental and AKT knockout HCT116 colon cancer cells (AKT1−/−, AKT2−/−, and AKT1/AKT2 DKO) [22]. HCT116 cells express AKT1 and AKT2 but not AKT3 [22]. We validated antibodies specific for AKT1, pAKT1 Ser473, AKT2, and pAKT2 Ser474 with good specificity and sensitivity. The AKT1 and pAKT1 Ser473 antibodies did not cross-react with AKT2 in AKT1−/− cells, and the AKT2 and pAKT2 Ser474 antibodies did not cross-react with AKT1 in AKT2−/− cells (Fig. 1a, left panel). A pan-AKT antibody and a phospho-AKT antibody (designed as pAKT Ser473/4 for AKT phosphorylation in the hydrophobic domain) recognized both AKT isoforms (Fig. 1a, left panel). AKT1 is the predominant isoform harboring hydrophobic domain phosphorylation, consistent with our previous data showing that pAKT1 at Ser473 accounts for the vast majority of total AKT hydrophobic domain phosphorylation in HCT116 cells [23].

We transfected HCT116 DKO cells to express AKT3 to further examine specificities of the antibodies. The isoform-specific antibodies for AKT1 and AKT2 did not cross-react with AKT3, except for a weak signal with pAKT2 Ser474 (Fig. 1a, right panel). The pan antibodies for AKT or pAKT recognized AKT3 (Fig. 1a, right panel). Because no phospho-AKT3 antibody is available at this time, we therefore focused on isoform-specific expression and phosphorylation of AKT1 and AKT2, the two ubiquitously expressed AKT isoforms [24].

We then assessed the AKT antibodies on the RPPA platform for specificity, quantification, and sensitivity (dynamic range) using lysates from 211 cell lines. RPPA signatures of protein and phospho-protein levels of AKT1, AKT2, and pan-AKT are summarized in Fig. 1b. To validate the RPPA results for the four AKT isoform-specific antibodies, we performed western blotting for cell lines expressing a range of levels of each AKT isoform (Fig. 1c). The levels of AKT1, phospho-AKT1 (pAKT1) Ser473, AKT2, and pAKT2 Ser474 detected by RPPA were highly correlated with those detected by western blotting (Fig. 1d, Additional file 2 a-c), indicating that the RPPA data collected for AKT isoforms were of sufficiently high quality for analyzing isoform-specific expression and activation of AKT in these cell lines.

### Coordinate phosphorylation, but not expression, of AKT1 and AKT2

**Fig. 2:**
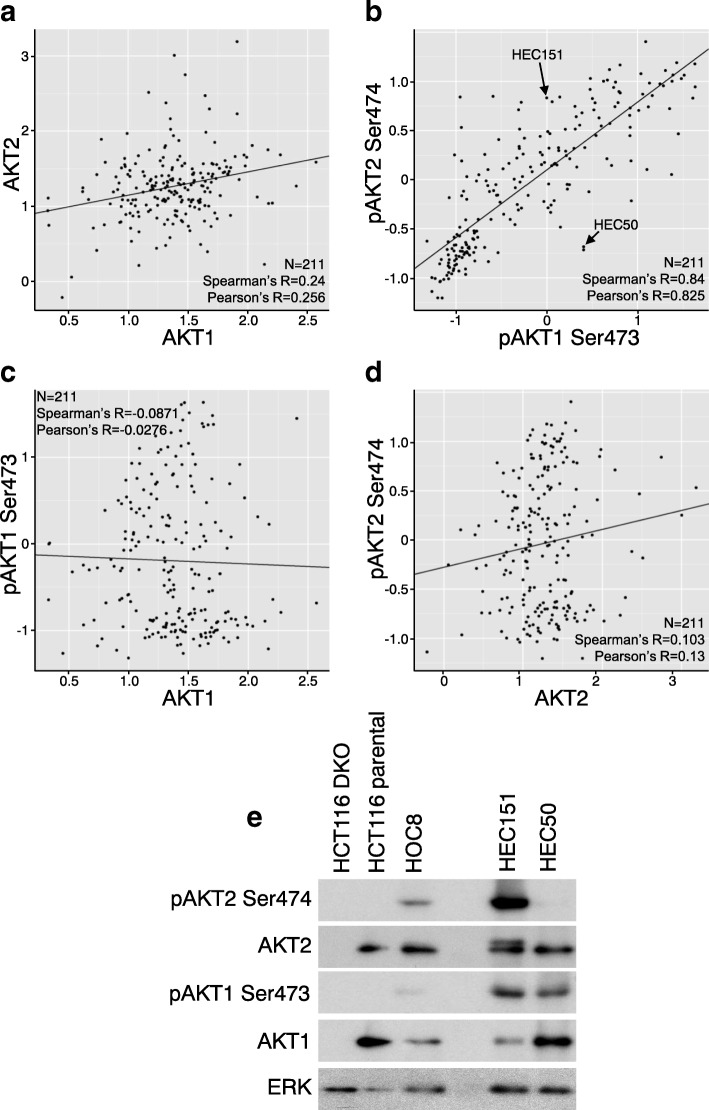
Differential AKT1 and AKT2 expression and activation across cell lines a-d Correlations of protein or phospho-protein levels of AKT1 and AKT2. The 211 cell lines were cultured in MD Anderson RPPA core. RPPA data for 211 cell lines were analyzed for correlations between AKT1 and AKT2 (**a**), pAKT1 Ser473 and pAKT2 Ser474 (**b**), AKT1 and pAKT1 Ser473 (**c**), and AKT2 and pAKT2 Ser474 (**d**). Spearman rank correlation coefficient and Pearson correlation coefficient are presented. Arrows in (**b**) designate HEC151 and HEC50 cells that were further analyzed. **e** Protein and phospho-protein levels of AKT1 and AKT2 in HEC151 and HEC50 cells. Two endometrial adenocarcinoma cell lines (HEC151 and HEC50) and other cell lines including HCT116 DKO, parental HCT116, and HOC8 were cultured in complete medium. Lysates (30 μg/lane) were resolved in 10% SDS PAGE. Antibodies for each blot are listed to the left

We then analyzed protein and phospho-protein levels of AKT1 and AKT2 in the 211 cell line set. AKT1 and AKT2 protein levels did not correlate (Fig. 2a), indicating that the expression levels of these two isoforms were independently regulated. Similarly, mRNA levels of AKT1 and AKT2 showed no correlation (Additional file 3 a). Both AKT1 and AKT2 protein levels only weakly correlated with their corresponding mRNA levels (Additional file 3 b, c), suggesting that mechanisms in addition to translation regulate AKT protein levels.

Phosphorylation in the hydrophobic domain of AKT (Ser473 of AKT1 or Ser474 of AKT2) is required for optimal activation and reflects the activation levels of AKT [25]. However, AKT isoform-specific phosphorylation in the hydrophobic domain has not been systematically characterized. In the 211 cell lines, pAKT1 Ser473 and pAKT2 Ser474 phosphorylation levels showed a significant correlation in the majority of cells (Fig. 2b), indicating that both AKT isoforms were similarly phosphorylated in most cases. However, a subset of cell lines showed high phosphorylation of one of the isoforms without concordant phosphorylation of the other isoform (Fig. 2b). Cell lines in the upper-left region of fig. 2b, i.e., KLE, TUOC1, PANC1, ARK2, and HEC151, showed relatively higher pAKT2 Ser474 than pAKT1 Ser473. Cell lines in the lower-right region, i.e., HCC2218B1M, HEC88NU, and HEC50, showed relatively higher pAKT1 Ser473 than pAKT2 Ser474. Phosphorylation of AKT1 or AKT2 did not correlate with their corresponding protein levels (Fig. 2c, d), indicating that AKT isoforms were not phosphorylated in proportion to their expression.

RPPA showed that two endometrial adenocarcinoma cell lines, HEC151 and HEC50, possessed similar pAKT1 Ser473 levels but markedly different pAKT2 Ser474 levels (Fig. 2b), suggesting that AKT isoforms were differentially phosphorylated in these cells. Western blotting validated the RPPA finding that pAKT2 Ser474 was much higher in HEC151 than in HEC50, although AKT2 protein levels were similar between the two cell lines (Fig. 2e). AKT2 protein ran as double bands in HEC151 but as a single band in HEC50 (Fig. 2e). The upper band of AKT2 in HEC151 likely represented phosphorylated AKT2 wherein phosphorylation is known to decrease motility through SDS gels. The levels of pAKT1 Ser473 were similar between HEC151 and HEC50 (Fig. 2e), consistent with the RPPA results (Fig. 2b). Taken together, these data indicate that AKT1 and AKT2 can be differentially phosphorylated in specific cells. HEC151 harbors PIK3CA and PTEN mutations, which are different from the mutations in HEC50 (e.g., PIK3R1 and KRAS) [26]. The extent to which these cell-specific mutations contribute to AKT isoform-specific phosphorylation warrants further study.

### Differential AKT1 and AKT2 expression and phosphorylation in different cancer lineages

**Fig. 3:**
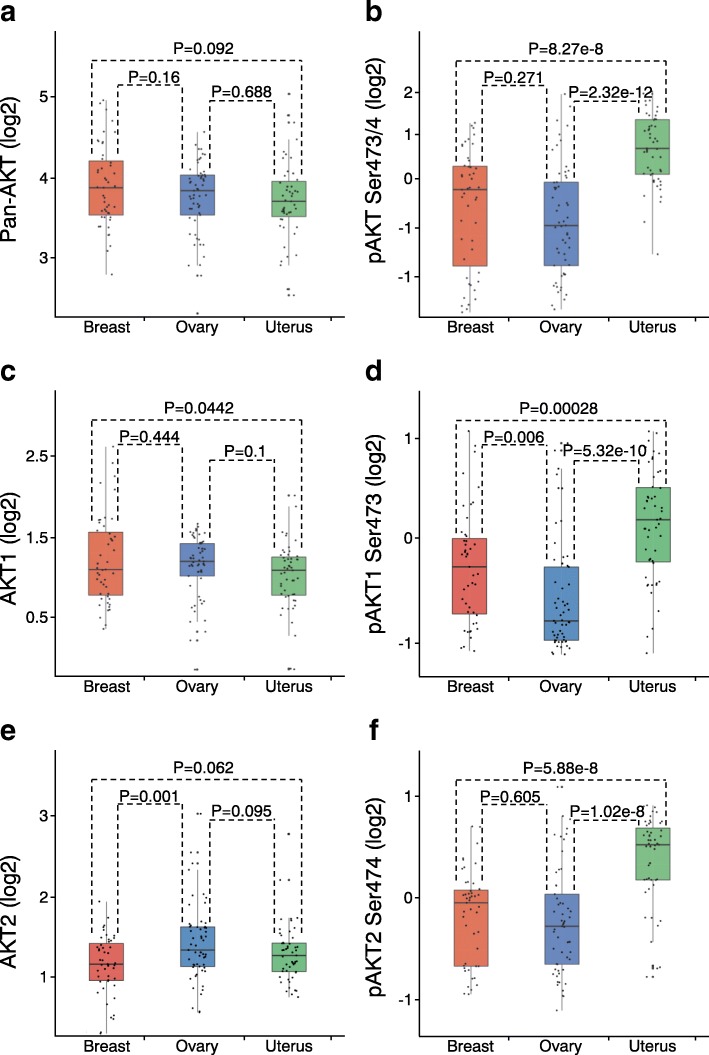
Differential AKT1 and AKT2 expression and activation across cancer lineages **a***,* **b** Box plots of protein (**a**) or phospho-protein (**b**) levels of pan-AKT by cancer lineage. *P* values by t-test on log-transformed values. Box plots represent 5, 25, 50, 75, and 95%. **c, d** Box plots of protein (**c**) or phospho-protein (**d**) levels of AKT1. **e, f** Box plots of protein (**e**) or phospho-protein (**f**) levels of AKT2

We compared protein and phospho-protein levels of AKT1 and AKT2 across different cancer lineages. Most of the cell lines (154 of 211) were from breast [47], ovarian [56], and uterine cancers [51]. Within each of these cancer lineages, a wide range of protein and phospho-protein levels of specific AKT isoforms was observed (Fig. 3). In contrast, median protein levels of pan-AKT did not differ significantly between the three lineages (Fig. 3a). However, AKT phosphorylation levels were significantly higher in uterine cancer than in breast or ovarian cancer (Fig. 3b), consistent with the high frequency of PTEN loss and PIK3CA activating mutations in uterine cancer lines [27]. The difference in AKT phosphorylation between breast and ovarian cancer was not statistically significant (Fig. 3b).

AKT1 was expressed at similar levels in breast and ovarian cancer lines (Fig. 3c). However, AKT1 phosphorylation levels were significantly higher in breast cancer than in ovarian cancer lines (Fig. 3d). AKT2 levels were higher in ovarian cancer lines than in the other two cancer types (Fig. 3e), consistent with previous studies showing that AKT2 is frequently amplified in ovarian cancer [28, 29]. The higher AKT2 levels in ovarian cancer compared with breast cancer did not result in higher AKT2 phosphorylation (Fig. 3f). Consistently with the results in Fig. 3b, phospho-protein levels of both AKT1 and AKT2 were high in uterine cancer (Fig. 3d, f).

### Differential AKT1 and AKT2 activation in different genetic backgrounds

**Fig. 4:**
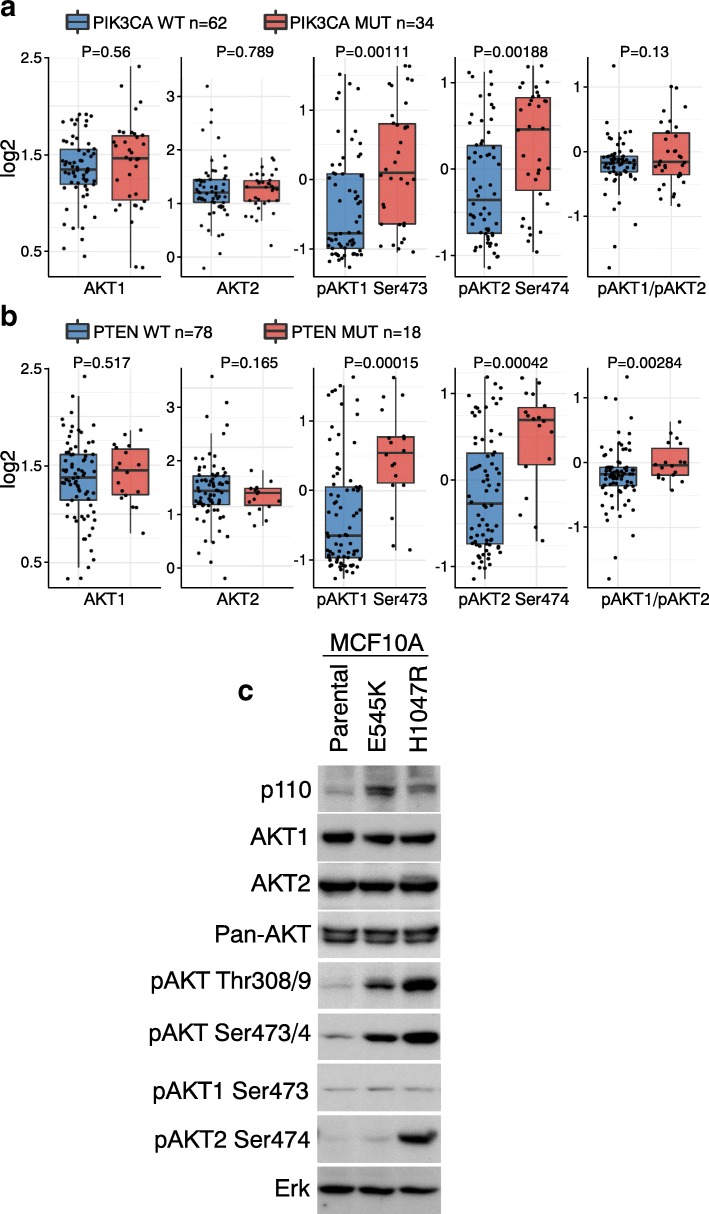
AKT isoform-specific activation in different genetic backgrounds **a** Box plots of protein or phospho-protein levels of AKT1 and AKT2 by status of PIK3CA (wild type [WT] versus mutation [MUT]). P values by t-test on log values. Box plots represent 5, 25, 50, 75, and 95%. **b** Box plots of protein or phospho-protein levels of AKT1 and AKT2 by status of PTEN. Box plots represent 5, 25, 50, 75, and 95%. **c** AKT isoform-specific activation by PIK3CA mutations. MCF10A Tet-on cells expressing PIK3CA E545K or H1047R mutations were cultured in complete medium. Cells were lysed in RIPA buffer with protease inhibitors and phosphatase inhibitors. Lysates (50 μg/lane) were resolved in 10% SDS PAGE. Antibodies for each blot are listed to the left. ERK immunoblotting shows equivalent loading

A variety of genetic alterations, such as amplification or mutation of PI3K and inactivation of PTEN, have been shown to hyperactivate AKT [3, 4]. However, the extent to which these genetic alterations preferentially activate specific AKT isoforms has not been as extensively studied. PIK3CA, the catalytic domain of PI3K, is an oncogene that is frequently mutated or amplified in cancer [30]. PTEN, the negative regulator of the PI3K/AKT pathway, is a tumor suppressor that is frequently mutated or deleted in cancer [31]. We analyzed the association between AKT1 or AKT2 activation with the mutation status of these two genes. PIK3CA mutations did not significantly affect AKT1 or AKT2 protein levels (Fig. 4a) but did markedly increase both pAKT1 Ser473 and pAKT2 Ser474. No AKT isoform specificity was observed in this association (Fig. 4a). PTEN mutations also markedly increased both pAKT1 Ser473 and pAKT2 Ser474 (Fig. 4b).

We further examined AKT isoform-specific phosphorylation by specific point mutations in PIK3CA. Hotspot mutations at E545 and H1047 in the hinge and catalytic domain respectively lead to higher activity of PI3K and to downstream AKT/mTOR activation [3, 32, 33]. In MCF10A cells, overexpression of PIK3CA E545K and H1047R did not alter AKT1, AKT2, or pan-AKT levels (Fig. 4c). However, H1047R increased phosphorylation at both the T-loop (pAKT Thr308/9) and the hydrophobic domain (pAKT Ser473/4) of AKT more than was observed with E545K (Fig. 4a), consistent with the concept that H1047R induces greater PI3K activation than E545K [34]. E545K and H1047R did not increase pAKT1 Ser473 (Fig. 4a). Serum and growth factors markedly increased pAKT1 Ser473 levels in MCF10A (Additional file 4), confirming that pAKT1 Ser473 levels can be induced in MCF10A, but not by the PIK3CA mutations. Interestingly, H1047R, but not E545K, markedly increased pAKT2 Ser474 (Fig. 4c), indicating that H1047R selectively activates AKT2. AKT3, which is expressed in MCF10A [35], likely contributed to overall AKT phosphorylation at the hydrophobic domain induced by E545K (Fig. 4c). Taken together, these results show that PIK3CA H1047R but not E545K selectively activates AKT2 but not AKT1 in MCF10A cells. There were too few PIK3CA hotspot mutations in the cell line set to allow analysis of effects of individual mutations. RPPA analysis on a larger sample set is warranted.

## Discussion

In this study, we used RPPA to analyze AKT isoform-specific expression and phosphorylation in 211 established cell lines using highly sensitive and specific antibodies. We further characterized AKT isoform-specific activation resulting from different PIK3CA mutations and PTEN loss. Our data revealed that AKT1 and AKT2 isoforms are differentially expressed and, in some cases, differentially phosphorylated in specific cancer lineages and genetic backgrounds. These findings should facilitate targeting AKT isoform-specific signaling in cancers.

AKT1 and AKT2 were for the most part coordinately phosphorylated; however, in certain cell lines, AKT1 and AKT2 are differentially phosphorylated, which reflects AKT activity. A variety of mechanisms have been proposed for AKT isoform-specific activation, including upstream kinase activity, phosphatases, post-translational modifications, substrates, and localization [13]. For example, depletion of INPP4B selectively activates AKT2 but not AKT1 in the endosomes of thyroid cancer cells [36]. In contrast, depletion of the inositol polyphosphate 5-phosphatase PIPP selectively activates AKT1 but not AKT2 [37]. We show in this study that AKT2 is preferentially activated by PIK3CA H1047R mutation in MCF10A cells. The mechanisms mediating AKT isoform-specific activation in response to this specific PIK3CA mutation are presently unknown, but the fact that another mutation in PIK3CA, E545K did not elicit the same response in MCF10A cells suggests subtle alterations in the structural features of PIK3CA can result in major shifts in cellular signaling.

PIK3CA mutations have been extensively characterized for downstream signaling activation in different genetic background [38–40]. We demonstrated that the PIK3CA hotspot mutation H1047R, but not E545K, selectively activates AKT2 but not AKT1 in MCF10A cells. The H1047R mutation has been shown to promote MCF10A cell invasion [34], consistent with observations that AKT2 is a driver of cell motility and invasion [9, 17, 18]. With this in mind, the H1047R mutation might prove to be a useful biomarker for AKT2 isoform activation in cancer patients with metastasis. We did not observe AKT isoform-specific activation when all PIK3CA mutations were analyzed as a pool (Fig. 4a), potentially because different mutations have different proclivities for activating specific AKT isoforms. The sample set was too small to analyze single mutations. In addition, we note that AKT isoform-specific activation is likely cell type- and context-dependent.

We focused on AKT1 and AKT2 in this study. However, AKT3 is also emerging as a critical driver and therapeutic target in a subset of cancer lineages. For example, AKT3 is frequently amplified and overexpressed in triple-negative breast cancer [35] and glioma [41]. AKT3 silencing inhibits spheroid and xenograft growth [35]. Fusion gene MAGI3-AKT3 also is enriched in triple-negative breast cancer [42]. AKT3 isoform-specific expression and activation warrant further study once pAKT3 antibodies are available with the required high specificity for this AKT isoform.

We have characterized AKT1 and AKT2 isoform-specific expression and phosphorylation in multiple cell lines. The next step will be to extend the studies to patients to identify biomarkers for AKT isoform-specific activation and targeting.

## Conclusions

In summary, we identified differential expression and phosphorylation of AKT1 and AKT2 isoforms in different cancer lineages and genetic backgrounds, including the selective activation by PIK3CA hotspot mutation H1047R of AKT2 but not AKT1. Our results advance our understanding of AKT isoform-specific signaling and facilitate the development of therapeutic approaches to targeting AKT isoforms in cancer.

## Additional files

Additional file 1: 211 cell lines used in RPPA analysis. (XLSX 17 kb)

Additional file 2: Validation of AKT isoform-specific antibodies by RPPA (*a*) Validation of an antibody of pAKT1 Ser473 for RPPA. Lysates of 211 cell lines were analyzed by RPPA in triplicate using the antibody of pAKT1 Ser473. Selected cell lines expressing pAKT1 Ser473 at various levels per RPPA data are shown in the bar graph with standard deviations as error bars. pAKT1 Ser473 levels in the selected cell lines were examined by western blotting. ERK immunoblotting was used as a loading indicator. Scanning densitometric values for pAKT1 Ser473 western blotting were obtained using ImageJ software (version 1.46r; National Institutes of Health, Bethesda, MD). Western data of pAKT1 Ser473 are presented as ln-transformed densitometric values. The correlation coefficient between signals derived from RPPA and Western blotting is shown. (*b*) Validation of an antibody of AKT2 for RPPA as in (*a*). (*c*) Validation of an antibody of pAKT2 Ser474 for RPPA as in (*a*). (PDF 84 kb)

Additional file 3: Differential AKT1 and AKT2 expression and activation across cell lines (*a-c*) Correlations of mRNA or protein levels of AKT1 and AKT2. One hundred fourteen of 211 cell lines with mRNA data were analyzed for correlations between mRNA levels of AKT1 and AKT2 (*a*), mRNA and protein levels of AKT1 (*b*), and mRNA and protein levels of AKT2 (*c*). Spearman rank correlation coefficient and Pearson correlation coefficient are presented. (PDF 144 kb)

Additional file 4: Induction of pAKT1 Ser473 in MCF10A cells MCF10A cells were starved in serum-free medium overnight. Serum-free medium was replaced or not replaced with complete medium containing horse serum (5%), EGF (20 ng/ml), insulin (10 μg/ml), Hydrocortisone (0.5 mg/ml), and Cholera Toxin (100 ng/ml) for 15 min. Cells were lysed in RIPA buffer with protease inhibitors and phosphatase inhibitors. Lysates (50 μg/lane) were resolved in 10% SDS PAGE. Antibodies for each blot are listed to the left. ERK immunoblotting showed equivalent loading. (PDF 14 kb)

## Abbreviations

DKO: Double knockout
INPP4B: Inositol polyphosphate-4-phosphatase type II
pAKT1: Phospho-AKT1
pAKT2: Phospho-AKT2
PI3K: Phosphatidylinositol 3-kinase
PTEN: Phosphatase and tensin homolog
RIPA: Radioimmunoprecipitation assay
RPPA: Reverse-phase protein array
