# Supplementary figures and images for: AKT isoform-specific expression and activation across cancer lineages
Source: BMC Cancer. 2018 Jul 16;18:742. doi: 10.1186/s12885-018-4654-5 (PMC6048698; doi:10.1186/s12885-018-4654-5)

## Additional file 2

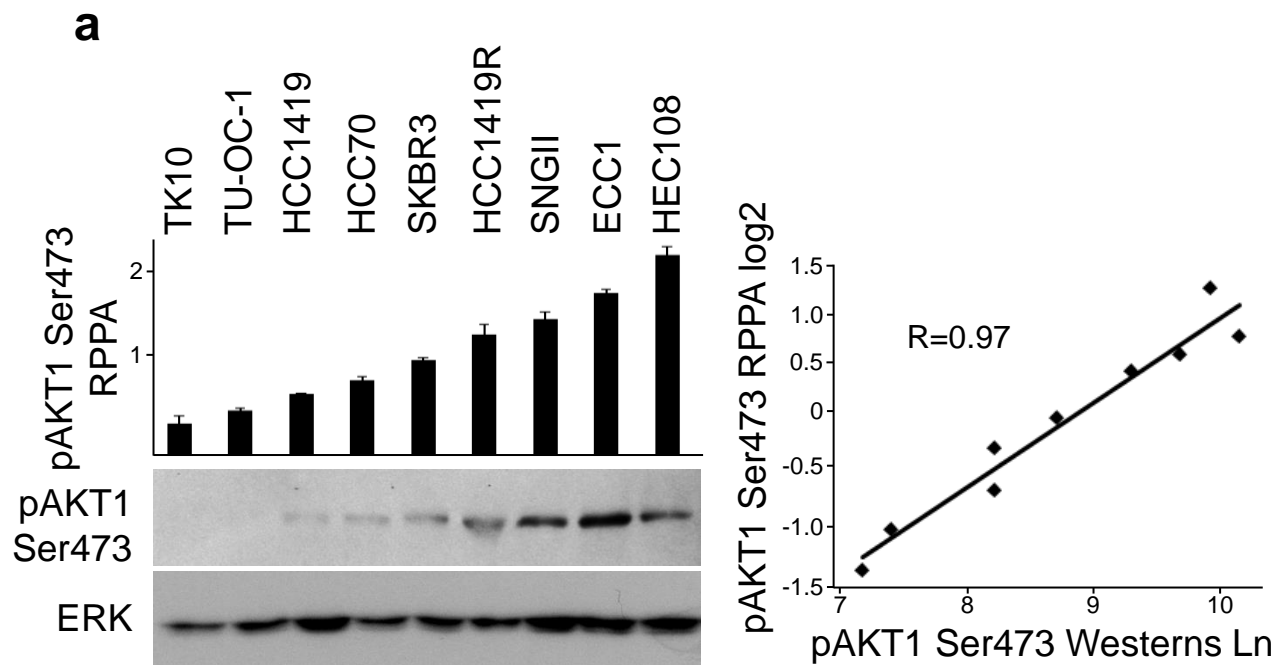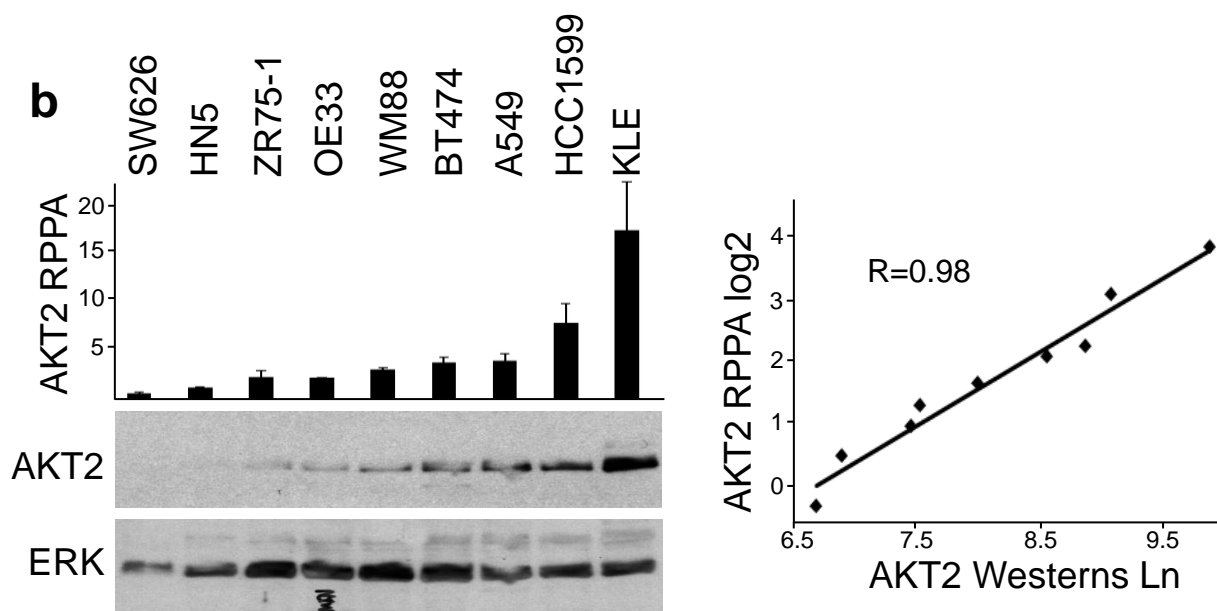

## Additional file 2

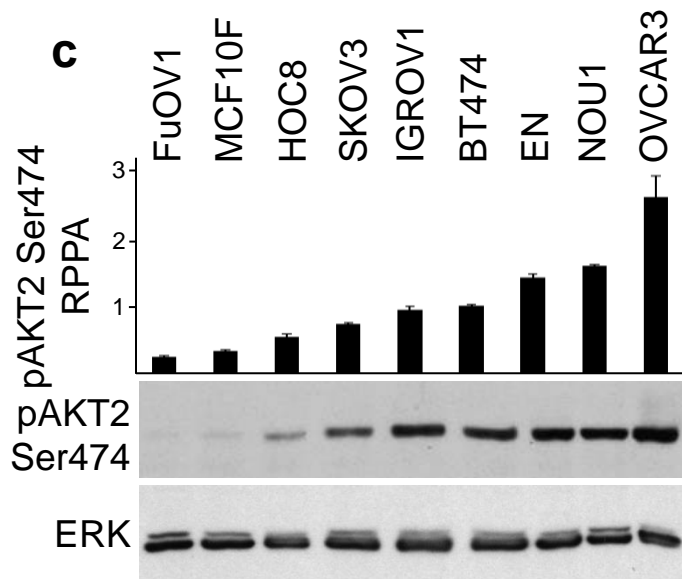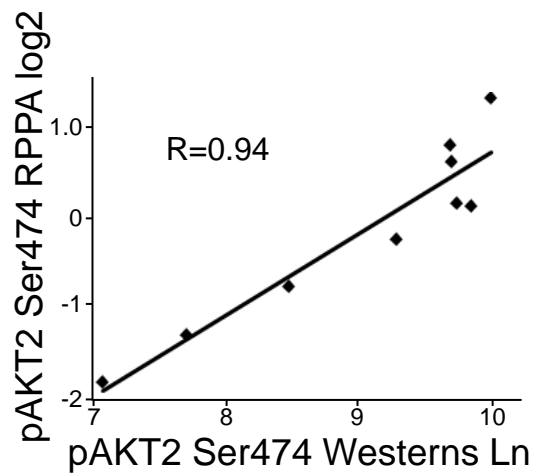

Supplement: Supplementary file 2 — Validation of AKT isoform-specific antibodies by RPPA (a) Validation of an antibody of pAKT1 Ser473 for RPPA. Lysates of 211 cell lines were analyzed by RPPA in triplicate using the antibody of pAKT1 Ser473. Selected cell lines expressing pAKT1 Ser473 at various levels per RPPA data are shown in the bar graph with standard deviations as error bars. pAKT1 Ser473 levels in the selected cell lines were examined by western blotting. ERK immunoblotting was used as a loading indicator. Scanning densitometric values for pAKT1 Ser473 western blotting were obtained using ImageJ software (version 1.46r; National Institutes of Health, Bethesda, MD). Western data of pAKT1 Ser473 are presented as ln-transformed densitometric values. The correlation coefficient between signals derived from RPPA and Western blotting is shown. (b) Validation of an antibody of AKT2 for RPPA as in (a). (c) Validation of an antibody of pAKT2 Ser474 for RPPA as in (a). (PDF 84 kb) [file 12885_2018_4654_MOESM2_ESM.pdf]

# Additional file 3

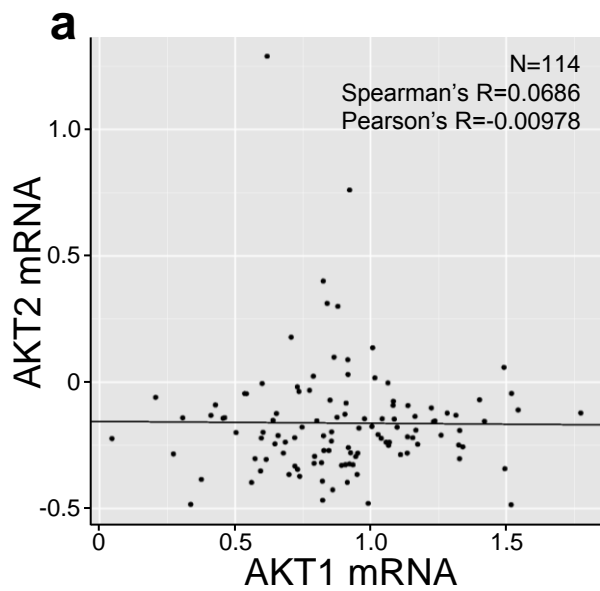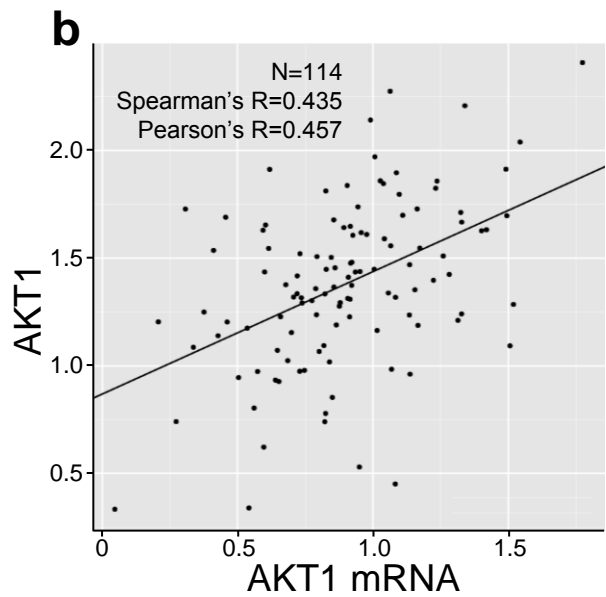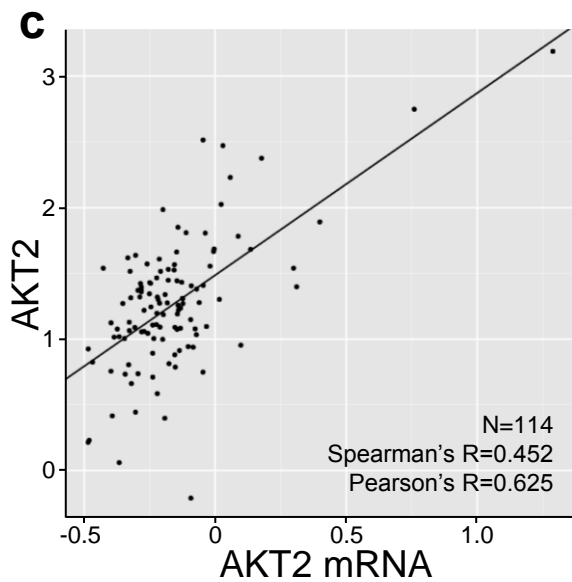

Supplement: Supplementary file 3 — Differential AKT1 and AKT2 expression and activation across cell lines (a-c) Correlations of mRNA or protein levels of AKT1 and AKT2. One hundred fourteen of 211 cell lines with mRNA data were analyzed for correlations between mRNA levels of AKT1 and AKT2 (a), mRNA and protein levels of AKT1 (b), and mRNA and protein levels of AKT2 (c). Spearman rank correlation coefficient and Pearson correlation coefficient are presented. (PDF 144 kb) [file 12885_2018_4654_MOESM3_ESM.pdf]

## Additional file 4

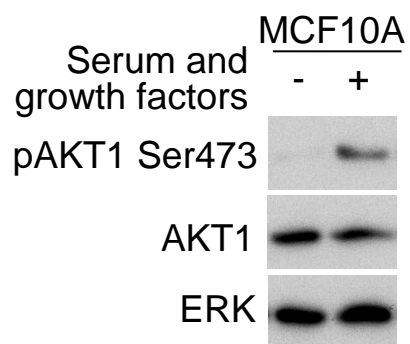

Supplement: Supplementary file 4 — Induction of pAKT1 Ser473 in MCF10A cells MCF10A cells were starved in serum-free medium overnight. Serum-free medium was replaced or not replaced with complete medium containing horse serum (5%), EGF (20 ng/ml), insulin (10 μg/ml), Hydrocortisone (0.5 mg/ml), and Cholera Toxin (100 ng/ml) for 15 min. Cells were lysed in RIPA buffer with protease inhibitors and phosphatase inhibitors. Lysates (50 μg/lane) were resolved in 10% SDS PAGE. Antibodies for each blot are listed to the left. ERK immunoblotting showed equivalent loading. (PDF 14 kb) [file 12885_2018_4654_MOESM4_ESM.pdf]
